# Process Modeling and Simulation of Tableting—An Agent-Based Simulation Methodology for Direct Compression

**DOI:** 10.3390/pharmaceutics13070996

**Published:** 2021-06-30

**Authors:** Niels Lasse Martin, Ann Kathrin Schomberg, Jan Henrik Finke, Tim Gyung-min Abraham, Arno Kwade, Christoph Herrmann

**Affiliations:** 1 Institute of Machine Tools and Production Technology (IWF), Technische Universität Braunschweig, Langer Kamp 19b, 38106 Braunschweig, Germany; t.abraham@tu-bs.de (T.A.); c.herrmann@tu-bs.de (C.H.); 2 Center of Pharmaceutical Engineering (PVZ), Technische Universität Braunschweig, Franz-Liszt-Str. 35A, 38106 Braunschweig, Germany; jan.finke@tu-bs.de (J.H.F.); a.kwade@tu-bs.de (A.K.); 3 Institute for Particle Technology (iPAT), Technische Universität Braunschweig, Volkmaroder Str. 5, 38104 Braunschweig, Germany

**Keywords:** agent-based modeling and simulation, process modeling and simulation, tableting, product structures and characteristics

## Abstract

In pharmaceutical manufacturing, the utmost aim is reliably producing high quality products. Simulation approaches allow virtual experiments of processes in the planning phase and the implementation of digital twins in operation. The industrial processing of active pharmaceutical ingredients (APIs) into tablets requires the combination of discrete and continuous sub-processes with complex interdependencies regarding the material structures and characteristics. The API and excipients are mixed, granulated if required, and subsequently tableted. Thereby, the structure as well as the properties of the intermediate and final product are influenced by the raw materials, the parametrized processes and environmental conditions, which are subject to certain fluctuations. In this study, for the first time, an agent-based simulation model is presented, which enables the prediction, tracking, and tracing of resulting structures and properties of the intermediates of an industrial tableting process. Therefore, the methodology for the identification and development of product and process agents in an agent-based simulation is shown. Implemented physical models describe the impact of process parameters on material structures. The tablet production with a pilot scale rotary press is experimentally characterized to provide calibration and validation data. Finally, the simulation results, predicting the final structures, are compared to the experimental data.

## 1. Introduction

The landscape of pharmaceutical processes and their control is and will further be changing in the coming decades. The adoption of quality by design (QbD) concepts and process analytical technology (PAT) approaches will be fostered, and classical batch production will be superseded by continuous pharmaceutical processes as well. The latter is getting more and more established in pharmaceutical industries due to its high potential in agility, flexibility, cost, and robustness and therefore ensuring a continuous supply of high-quality drugs [1]. Nevertheless, continuous as well as batch processes can be represented in simulation models, allowing the investigation of parameter changes and the resulting impact on the products. At the moment, and in the transition towards a continuous production in pharmaceutics, the process chains consist of combined batch and continuous processes, where each process has a significant impact on intermediate and final product structures and characteristics. Therefore, process chain models may need to represent batch as well as continuous processes.

Until now, the verification of final product quality is mainly ensured by offline batch acceptance sampling after nearly every process step [2]. In case of process deviations, methodologies forecasting the final product quality or even feed-forward oriented control strategies for process parameter adaption are mostly missing. Hence, this gap often causes batch loss and imposes high cost.

**Figure 1:**
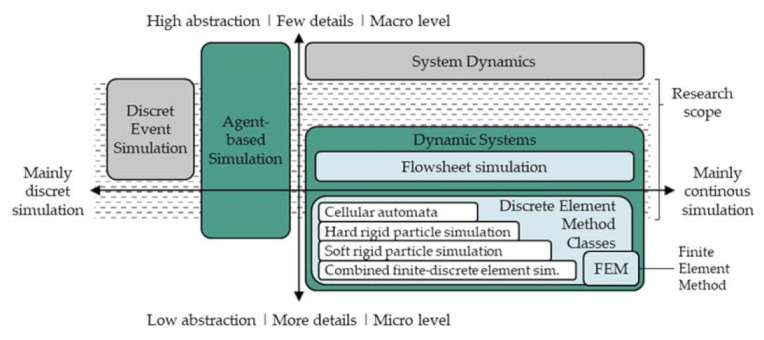
Typical dynamic simulation approaches for the tableting process mapped on the simulation modeling paradigms (following [4,5,6]).

Dynamic simulation approaches allow for the forecasting of processes and their process chains and, when combined with real world data, data-driven, knowledge-based control of these processes [3]. In the planning phase, forecasting the resulting product structures and characteristics of the planned process chain is possible. In the operation phase and based on proper simulation approaches, deviations can be compensated for by adjusting the parameters of upcoming process steps, adapting treatment times, or repeating or adding further steps. Furthermore, simulation approaches allow the analysis of complex interdependencies and are therefore useful to generate process knowledge. Four typical simulation paradigms can be distinguished in dynamic simulation modeling [4] and are shown in Figure 1. Depending on the abstraction level of the model and the change of process parameters over time (continuous or discrete), different paradigms are more likely to be chosen. For example, models with a high degree of detail and a continuous parameter change are likely to be modelled using the dynamic systems paradigm. 

Discrete event simulations (DES) are widely used for modeling complex networks such as Petri nets, which do not require continuous parameter changes but are very helpful for the determination of key performance indicators of process chains. System dynamics (SD) models describe the system behavior with few details of the entities on a high abstraction level, and are often used for complex social or political systems [5].

For simulating a tableting process or sub-process, the common approach is the use of dynamic systems simulations such as flowsheet simulations (FSS). Furthermore, discrete element methods (DEM) are used, which can further be classified depending on their level of detail. This scale can reach from cellular automata to combined finite-discrete element simulation as illustrated in Figure 1. A detailed description for those methods can be found in [6]. Simulations built upon the finite element method (FEM) are used to model particle deformation behavior under external stresses and the propagation of stresses within particles. Cellular automata approaches model the interaction with neighboring particle groups rather than the particles themselves, while combined finite-discrete element approaches model particle deformation, interaction with other particles, form and size in detail. FSS are mainly used to describe the change of properties of continuous phases (whereas solids are also considered continuous phases) and are well established in the process industry. Similar to the DES models, networks of entities and transitions are formed, representing process chains and aggregates, for example. The entities of a FSS network, so called nodes, are used to calculate output streams of given input streams according to physical circumstances. Due to the more complex description of solids, the use of FSS models for solid processes has only recently been investigated [7,8,9].

Those dynamic systems simulation approaches have already proven their value in simulating production processes and chains or detailed process behaviors, respectively. However, considering the different abstraction levels of the approaches, FSS lack the ability to determine distributed product properties and their influence on the process. In contrast to that, discrete element simulations lack the ability to simulate whole process chains due to high computational efforts [10]. In past research, there have been efforts to adapt FSS especially for particulate processes in order to model detailed product characteristics to overcome this gap. This resulted in a modular open source system covering diverse process units by using multidimensional distributed product structures [11]. Additionally, reduced order models allow the integration of originally highly detailed models in FSS approaches at low computational expense [12].

In comparison to the common dynamic systems modeling approaches, agent-based (AB) models allow both the ability to represent process chain behaviors and the ability to determine heterogenic product properties as well as their interdependencies with the processes and the consideration of different detail levels [4,13]. Therefore, AB models could be used to simulate intermediate product changes and the effects of those changes on the process and vice versa. Furthermore, AB models are able to represent cellular automata [13]. This enables the simulation of interrelations between neighboring materials and processes, a typical discrete element simulation characteristic [6]. Therefore, AB simulations helps to gain a better understanding of process chains, sub-processes as well as product properties, and represents a practical alternative to the existing simulation approaches. Especially for considering not only single processes, but the complete process chain of a pharmaceutical production, AB approaches have advantages in traceability and the transfer of product characteristics for specific entities.

The overall aim in this research is to evaluate processes and entire process chains in the planning phase and to support process and process chain improvements during operation with respect to the quality of pharmaceutical products using simulations. AB models are capable of integrating the advantages of the common dynamic systems approaches. Consequently, an AB model on the tableting process in a rotary press is introduced, providing the basis for further research. The process step of tableting is chosen to demonstrate the applicability of the approach, which is, however, a generic approach that can be applied virtually on other process steps in the process chain. It highlights the necessity of careful process description and analysis (Section 2), setup of an AB model framework for a rotary press (Section 3), model identification, development and integration into this simulation approach, and its application and comparison to experimental results (Section 4), demonstrating its capability of determining product characteristics from parameter settings as well as tracing discrete product entities and material structures.

## 2. Tableting and Its Simulation

The diversity of particulate pharmaceutical products is high, reaching from powders to suspensions with numerous therapeutical applications and production processes. Due to its market share, the tablet is probably the most prominent particulate pharmaceutical product and there are several process routes for the production of tablets.

**Figure 2:**
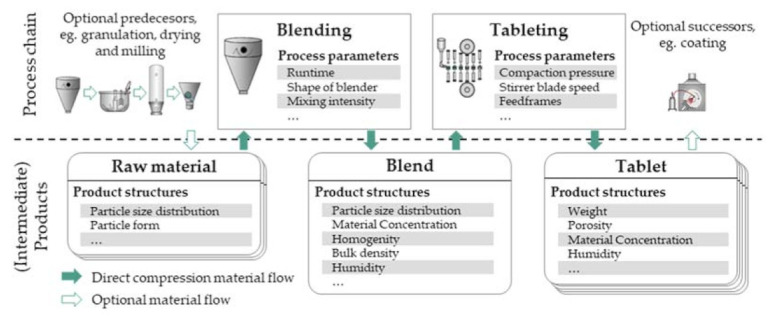
Direct compression process chain with exemplary (intermediate) product structures.

Direct compression, only comprising blending of raw materials and tableting, is the preferred process route for the tableting process, as very few process steps and handling are required and the powder materials are exposed to low temperatures, low moisture stresses, and short process times. The process parameters and the product structures for the direct compression process chain are illustrated and highlighted in Figure 2. This process chain covers several of the above-mentioned challenges, such as the combination of batch and continuous processes to produce a discrete final product or the variation of intermediate product structures resulting in critical quality attributes (CQAs) of the final product.

In addition, the applicability of direct compression is limited due to the high demands on the formulation regarding good flowability and low segregation tendencies. Depending on the properties and structure of API particles, the basic process chain of direct compression needs necessarily to be extended by different possible process steps, which are for example different granulation processes or subsequent tablet coating. Accordingly, the combined simulation of all processes for direct compression and the inclusion of granulation and coating will be the subject of future research. This research treats the direct compression process chain with a special focus on the tableting process.

### 2.1. Tableting Process

**Figure 3:**
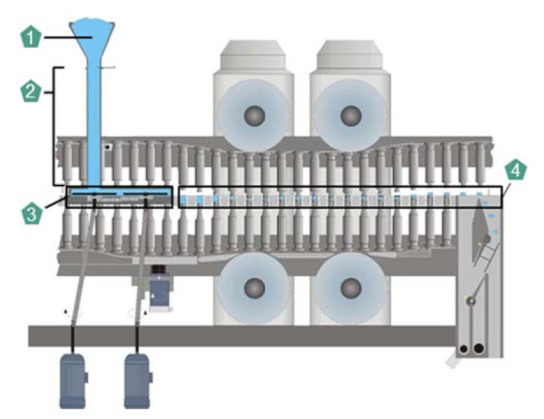
Rotary press components (provided by Korsch AG).

In the addressed tableting process, the process parameters and the preliminary product properties determine the intermediate product structures during the tableting process and consequently the final tablet properties. The tablet quality is often described by CQAs. As this case study focuses on the tablet structure depending on the excipient composition, the mass fraction of the respective excipient, tablet weight, porosity, and tensile strength are selected as CQAs. The porosity influences the disintegration time of the tablet and depends on the compression stress and the excipient properties. Additionally, a sufficient tensile strength is required to enable the handling and further processing of the tablets. The tableting process step exerts the highest influence on these tablet CQAs for the direct compression process chain. Therefore, the tablet production on a rotary press in pilot-scale was selected to be the simulative and experimental setup for this study. In Figure 3, the relevant components of a rotary press are shown. It consists of

1. a hopper containing the blend,
2. a filling pipe transporting the blend into the feed frame,
3. a feed frame equipped with one to three rotating paddle wheels with several stirring blades, transporting and filling the blend into the dies,
4. dies (and punches), passing the pre and main compression roller and the ejection mechanism. The main compression roller performs the compression of the powder in the die, leading to the formation of a tablet and the latter enables the ejection of the tablet from the die and out of the press.

The computational modeling of real-world processes allows better analysis and/or control of the processes. The modeling of the tableting process has already been described in the literature using different computational approaches, such as artificial intelligence [14,15], stochastic [16,17] and different simulation approaches (e.g., [18]), of which the existing FSS and DEM approaches are described in further detail below.

### 2.2. Existing Simulation Approaches and Agent-Based Simulation

Only a few approaches consider the complete direct compression scope. Simulation approaches using dynamic flow sheet approaches and considering the tableting process step in a direct compression are listed in Table 1. Compared to the low number of FSS approaches, several DEM and FEM approaches that consider the sub-process of the compression process exist.

#### 2.2.1. Dynamic Flowsheet Simulation Modeling

Boukouvala et al. use FSS modeling for the simulation of continuous tablet production via direct compression and compression after dry granulation. The tablet press is modelled in a very simplified manner using the popular Heckel equation in order to determine the porosity of the tablets. Therefore, the porosity of the final tablet is predominantly determined by the compaction force of the tablet press and material properties. Sub-processes such as the hopper, the filling pipe, or the feed frame are explicitly not considered, even though the conducted experiments show segregation in the feed frame. The input variables are varied, and a Monte Carlo simulation is applied to achieve multiple output evaluations to assess the sensitivity of the input parameters towards the output variable. This is carried out for different stages of the tablet production. The change of input variables over time is not explicitly considered [2].

In order to consider further CQAs, Boukouvala et al. have extended their first model with the aim of determining the tablet dissolution. The tablet dissolution is mainly determined by the tablet porosity, as well as composition and particle size distribution. For the tableting model, they used a Kawakita equation for modeling the final tablet porosity and the final tablet hardness is modelled as a function of the tablet relative density according to Kuentz and Leuenberger [19,20].

Rogers et al. extend the work of Boukouvala et al. [2,20], enabling the simulation of further CQAs such as the tensile strength [21], while Singh et al. even describe a control strategy for a multi-purpose continuous processing of pharmaceutical processes using the FSS model of Boukouvala et al. [9,20]. The dynamic FSS modeling approaches allow a good understanding and overview over the process chains and their parameters. Those parameters can even be controlled. However, the crucial components of FSS are the models for the individual processes and sub-processes. Those processes can be modeled to a certain degree of detail using semi-empirical or physical models. Comprehensive physical models that need microscale simulation, such as DEM, are not considered within the dynamic FSS, and therefore the determination of parameters for single products or product groups remains vague and could be improved [8].

#### 2.2.2. Discrete and Finite Element Modeling

The discrete element modeling approaches are rather used to describe specific sub-processes such as the compression [22,23,24,25] or the die filling [26,27,28,29,30,31,32]. The particles in a typical DEM approach are modeled rigidly and are mainly used for explanatory models of physical behavior rather than for engineering purposes, which is to some extend the approach of the Finite Element Method (FEM), allowing the consideration of stresses within the machine design [22,33,34]. Baroutaji et al. describe the development of a FEM simulating the tablet compression to analyze, for example, the density distributions and stress maps in tablets or the tooling geometry [22]. Lewis et al. describe the development of an efficient combined finite-discrete element method (CFDEM) in order to simulate the powder compression of the tableting process [23]. The authors modeled the particles involved in the compression, considering the particles’ individual shape, size, and size distribution. These approaches are modeled with a high level of detail of the particles and are useful for gaining a deeper process understanding, but due to time-consuming calculations, they are not appropriate for the simulation and control of process chains.

#### 2.2.3. Agent-Based Modeling

**Figure 4:**
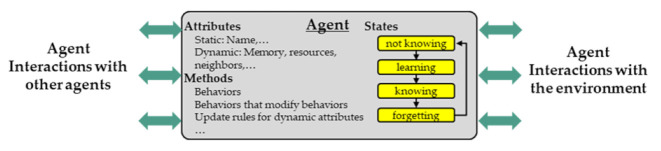
Typical agent structure and interactions (following [13]).

The structure of a typical AB model consists of agents, their relationships to other agents, and their environment. The agent’s own structure is shown in Figure 4.

The attributes of an agent can be static or dynamic, and each agent has a behavior that might change depending on rules adapting the behaviors. Such rules can be, for example, physical models such as the Heckel equation or they can depend on the state of the agent. The states are connected via transitions that follow specific rules before the next state is reached. Depending on the logic of the state, those transitions switch their behavior time dependently, at specific ratios, or via trigger. Those triggers can occur from the interactions with other agents or the agent’s environment. These and other aspects as well as use cases of AB modeling and simulation are well described in the literature [13,35,36].

The AB simulation paradigm was intended to simulate the dynamics of complex systems consisting of populations of autonomous, interacting agents or components. Nowadays, AB approaches are more and more common in production engineering [36]. AB simulation in particular can be used to describe the interactions of machines and (intermediate) products represented by individual agents [37].

To the authors’ knowledge, AB simulation has not been used for pharmaceutical processes generally or tableting specifically. AB simulation enables the analysis of the development for specific product characteristics over the entire process chain, which is an advantage in comparison to the DEM and FSS. Here, processes can be adapted in the process of product development. Furthermore, the product quality can be controlled during the production phase. In addition, discrete and continuous processes and sub-processes can be considered together, which enhances the process understanding and is essential in the transition to continuous pharmaceutical production.

## 3. Agent-Based Simulation Model for the Tableting Process

For the development of an AB model of the tableting process, a thorough understanding of the sub-processes is required. Process models that describe the relationship between process parameters and product structures are necessary. A typical approach of modeling agents is outlined by Macal and North [35], consisting of the following three interlinked steps:

I. the identification of agents, agent groups, and their attributes,
II. the specification of the agent’s behavior and
III. identifying the agent’s interactions.

Following these three steps, the AB model was derived. For the derivation of the model, the pilot-scale rotary press XL 100 (KORSCH AG, Berlin, Germany) was considered, although the derived AB model is able to simulate any rotary press. A feed frame with one rotating paddle wheel with twelve stirring blades was used for the model setup as described below.

### 3.1. Step I|Identification of Agents, Agent Groups and Their Attributes

**Figure 5:**
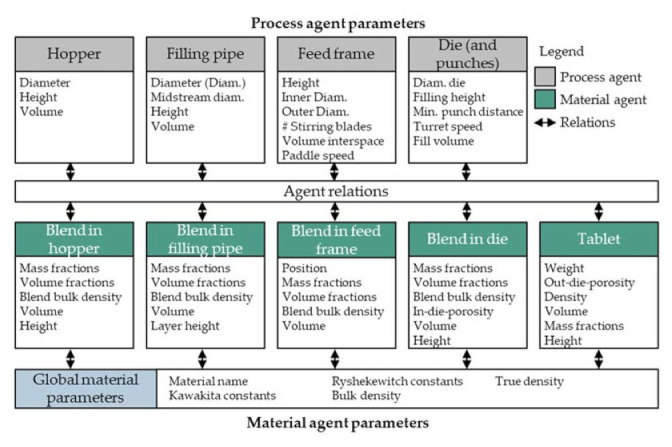
Existing model agents and their required parameters.

Two different agent types are introduced and shown in Figure 5; process agents and material agents. Process agents can be identified by determining the processes that have an individual behavior and a main impact on the final product structure. Considering the above mentioned rotary press, the process agents can be identified as (A) the hopper, (B) the filling pipe, (C) the feed frame and (D) the die (and punches).

The identified material agents are the blend in the four different sub-process steps and especially the tablets at the end of the sub-process chain. Four different blend material agents are considered so as to better model the process-material behavior, the intermediate products, and the final tablet structures. The process parameters of the agents are listed in Figure 5. For the ease of understanding and the distinction of original processes and modeled agents, in the following all agents are written in *italics*.

**Figure 6:**
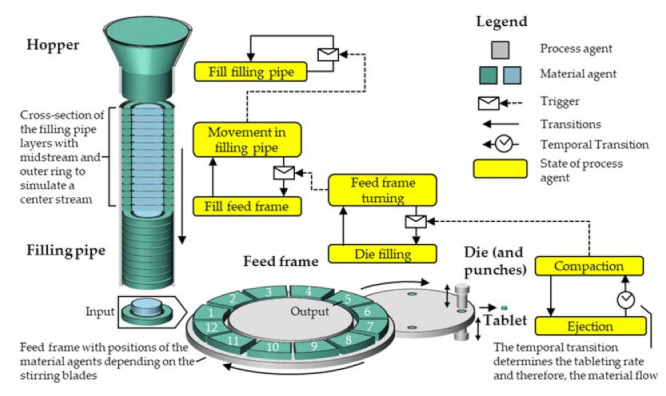
Schematic visualization of the process and material agents with the relevant states of the process agents.

As the CQAs of the final product are in the focus and the material flow rate is determined by the number and weight of tablets that are produced per time, the process chain and its parametrization is derived starting from the last material agent (*tablet*) and ending at the first process agent (*hopper*) to reasonably define the sizes and interactions. The agents in the agent group *tablet* are modeled as a passive data box, where the product structures and properties for each tablet are collected. Even though several dies are filled simultaneously on a rotary press, only one die is compressed at a time. Therefore, the authors choose to model the *blend in* *die* as a single agent representing alternatingly all dies of the rotary press. The twelve rotating stirrer blades on the paddle wheel in the feed frame transport the powder inside their respective interspaces. These interspaces are modeled as twelve compartments, each represented by one *blend in feed frame* agent. In order to simulate the powder flow within the filling pipe, a group of agents is defined as the *blend in filling pipe.* A flow profile inside the pipe with slower flow close to the wall compared to the middle is expected. Therefore, the agent group consists of two agent types–the *midstream*, representing the higher velocities in a central circular region, and the *outer ring* (Figure 6), in which velocities are lower. Both types of agents possess the same height in every vertical position, forming layers within the *filling pipe*, each containing a *midstream* and an *outer ring*. The *midstream* agents travel faster than the outer ring agents by a constant velocity difference.

In the state of equilibrium, the inflowing and outflowing powder streams in the feed frame and the filling pipe are equal in volume. Thus, the volume of the powder agents entering the feed frame from the filling pipe equals the powder volume filled to the die—the dosing volume. Therefore, the summarized volumes of one *outer ring* and a defined number of *midstream blend in filling pipe* agents, dependent on the velocity difference, are equal to the dosing volume of the *die*. The *hopper* serves as a feed for the blend in this model and the *blend in hopper* volume is reduced according to the remaining blend in the hopper.

### 3.2. Step II|Specification of the Agents’ Behavior

For the specification of the agents’ behavior, the different states of the process agents were identified and are shown in a schematic overview for the tableting process in Figure 6. Furthermore, process knowledge of each state of the agent groups needs to be implemented in the AB model according to the agents’ behavior. In order to get the desired information, the process-structure relationships for the five process agents are described below.

#### 3.2.1. Hopper

The hopper is used as a reservoir for the blend, which is initially introduced to the tableting process. The *blend in hopper* agent’s structure is homogeneously distributed. Within the ‘fill filling pipe’ state, the structure of the *blend in hopper* is transferred into the newly generated *blend in filling pipe* agents. Thus, the mass fraction of the components inside the filling pipe is determined by the feed exiting the hopper. Segregation phenomena that may occur already in the hopper (e.g., during filling) will be the focus of future research work and are not included in this paper.

#### 3.2.2. Filling Pipe

Due to the flow profile inside the pipe, the composition changes over the height of the pipe as well as over time, resulting in a time-dependent composition entering the feed frame. The *blend in filling pipe* agents neither interact with agents in the horizontal nor in the stream wise direction. However, the AB approach is prepared to incorporate such segregation and distribution phenomena in future research. The transferred material of the *blend in filling pipe* agents is mixed with the remaining powder of the *blend in feed frame* agents.

#### 3.2.3. Feed Frame

The *feed frame* has twelve defined positions to which the *blend in feed frame* agents are clearly assigned (Figure 6). Inside the feed frame, a rotating paddle wheel transports the powder. In position 1, the interspaces are filled with powder from the *blend in filling pipe* agents. In the output position (5), the blend is filled into the die. The die is crossed by several feed frame paddles, so powder is filled from different *blend in feed frame agents* into the die with a descending proportion over time. Therefore, the product structures of the *blend in die* needs to be calculated according to the structures of the *blend in feed frames* and their filling proportions. The volume of the *blend in feed frame* is reduced accordingly. After each die filling, the *feed frame* agent rotates the *blend in feed frame* agents into a new position depending on the paddle speed. The mean bulk density during die filling can be calculated from the mass fraction *x_i_* of the individual *blend in feed frame* agents entering the die and the respective bulk density of the powder *ρ_bulk,i_*:

(1) $$\frac{1}{\rho_{fill,mix}}=\underset{i}{\sum }\frac{x_{i}}{\rho_{bulk,i}}$$

Based on the powder weight filled into the die, the tablet weight $m_{T}$ can be calculated considering the dosing height *h_dos_*:

(2) $$m_{T}=\pi \times {\left(\frac{D_{die}}{2}\right)}^{2}\times h_{dos}\times \rho_{fill,mix}$$

#### 3.2.4. Die (and Punches)

During the compression, the filling height is reduced to the compression height *h_min_*. For the simulations as well as for the experiments the compression height is set to a constant value and thus determines the developing compression stress. Consequently, the density of the *blend in die* agent *ρ_comp,mix_* changes during compression, which can be calculated from the minimal in-die height *h_min_* and the tablet weight *m_T_*:

(3) $$\rho_{comp,mix}=\frac{m_{T}}{\pi \times \left({\left(\frac{D_{die}}{2}\right)}^{2}\right)\times h_{min}\;\;}$$

The compression curve (porosity over compression stress) can be applied to determine the resulting stress (and calculate the respective force) that is applied to achieve the bespoke compression density $\rho_{P,max,mix}$. A wide range of models was developed, describing such compressibility curves by mathematical equations [38,39]. The most prominent compression model is the model of Heckel [40]. Further compressibility models were developed by Kawakita, Gurnham, Cooper, and Eaton, as well as Wünsch et al. [39,41,42,43]. However, for describing the compressibility behavior of powder blends with known but varying composition, an approach with an appropriate mixing rule is necessary. Busignies et al. presented a volume-additive approach to predict the tablet density of a formulation using the Kawakita model [44]. They transformed the classical equation into a model of the tablet density where *ρ*_0_*_,i_* describes the density at low pressure (here 20 MPa) and *a_i_* and *b_i_* are constants that need to be calibrated for the respective material:

(4) $$\frac{1}{\rho_{P,max,mix}}=\underset{i}{\sum }\frac{x_{i}}{\rho_{0,i}}\left(\frac{1+\left(1-a_{i}\right)\times b_{i}\times P}{\left(1+b_{i}\times P\right)}\right)$$

In this study, Equation (4) is converted to equal the compression stress *P* and solved via the *pq*-formula to gain the compression stress of a blend with two components.

From a process agent’s point of view, the ejection state is used to create a new *tablet* agent. After ejection, the tablet density changes due to the elastic recovery. Hirschberg et al. developed an approach to predict the out-die density *ρ_out-die_* based on the data of two tablets compressed at high and low compression stresses [45]. Therefore, the measured densities at maximum stress *ρ_P,max_*, at zero axial stress *ρ_P_*_,0_, and out-of-die *ρ_out-die_* are used. The change in density due to the instantaneous elastic recovery (Δ*ρ_in-die_* = *ρ_P,max_* − *ρ_P_*_,0_) is expected to increase linearly with the compression stress while the change due to the slow elastic recovery (Δ*ρ_slow_* = *ρ_P_*_,0_ − *ρ_out-die_*) is expected to be constant:

(5) $$\Delta \rho_{in-die}=k_{\Delta \rho_{instant}}\times P+l_{\Delta \rho_{instant}}$$

(6) $$\Delta \rho_{slow,\;ø}=\frac{\Delta \rho_{slow}\left(P_{1}\right)+\Delta \rho_{slow}(P_{2})}{2}$$

(7) $$\rho_{out-die}=\rho_{P,\;max}-\Delta \rho_{in-die}-\Delta \rho_{slow,ø}$$

In order to predict the out-die density for mixtures of the excipients with varying compositions, a mixing rule has to be introduced. For the mixtures, only the mass fraction and the in-die density at the apparent stress $\rho_{P,\;max,mix}$ are known, so the elastic recovery has to be described based on the data of the pure substances. Therefore, according to the volumetric approach of Busignies et al., the densities at the different states (*ρ_P,max_*, *ρ_P_*_,0_ and *ρ_out-die_*) for the high and low compression stress, investigated for the pure substances, are calculated for the mixtures (according to Equation (1)). Following this, the change in in-die density and out-die density (Equations (5) and (6)) can be calculated. Finally, the predicted out-die density results from the apparent in-die density at maximum stress *ρ_P,max_* minus the corrective function Δ*ρ_in-die_* at the respective compression stress and the corrective value Δ*ρ_slow_*_,ø_ (Equation (7)). Finally, the out-die density is corrected using the difference of the predicted values and the measured out-die densities, as suggested by Hirschberg et al.

The final tensile strength of the tablets dominantly depends, besides other influences, on the out-die porosity. In general, the lower the out-die porosity *ε_out-die_*, the higher the tensile strength *σ*. Ryshkewitch and Duckworth developed an exponential model to empirically describe this relationship [46]:

(8) $$\sigma =\sigma_{0}\times e^{-k_{b}\times \varepsilon_{out-die}}$$

The coefficient *σ*_0_ describes the strength of a nonporous body of the same material, while *k_b_* corresponds to the slope of the ln *σ* vs. *ε_out-die_* curve.

### 3.3. Step III|Identifying the Agents’ Interactions

**Figure 7:**
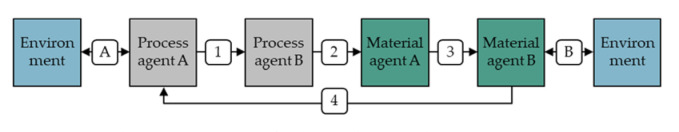
Exemplary agent interactions for this simulation model.

As described in Section 2.2, interactions in AB models can occur between agents (1–4) and between agents and their environment (A,B) as depicted in Figure 7. The latter, i.e., the environmental impacts on the process and the process impacts on the environment, have not been further considered even though aspects such as the humidity can have a significant impact on the product output. This research focuses on the interactions between agents. These interactions are modeled for connecting agents with the agents in their defined neighborhood and the dynamics that arise through this connection. Such interactions are mostly triggers (compare Section 2.1) for the transitions described in Step II.

As illustrated in Figure 7, interactions between process agents (1), between process and material agents (2), as well as vice versa (4), and between material agents (3) are considered in this model.

The process-process interactions have already been shown in Figure 6 and are marked via triggers. These triggers align the processes behaviors. Therefore, only if a trigger is sent, the trigger-receiving transition of the process agent is able to open the transition. This enables the agent to switch into the next state connected by the transition and show its specific behavior. One purpose of a trigger could be, that a process can send a trigger informing other processes that a specific behavior of a state has been completed. This occurs for example after the creation of a tablet. At that moment the *punch and die* agent interacts with the *feed frame* agent to allow the refill of the next die. Several process-material interactions are implemented in the model, e.g., the material agents’ volume depends on process agents’ parameters such as the volume of the *blend in pipe* agents, which is dependent on the volume of the *die* and the geometries of the *filling pipe*. The material-material interactions are necessary to transfer information from one material agent to another. This occurs every time a new material agent is created and obtains its attributes from corresponding agents. The same applies when material agents merge with other material agents and the product structures of both agents need to be assigned and calculated, respectively. Interactions between material agents occur in the filling processes of the filling pipe, the feed frame compartments, and the die. In those cases, a new blend density and mass fractions are calculated. The material-process interactions are relevant for the correct result of the final tablet properties. One of those cases is the calculation of the punch distance, which differs slightly from *h_min_* due to the elastic deformation of the rotary press depending on the applied compression stress resulting from the resistance against compression of the blend in the die.

An overview of the agents’ states, their respective behavior as well as interactions and the required calculations are listed in Table 2. Important process input parameters for the simulation are the minimal in-die height *h_min_*, the dosing height *h_dos_*, and the turret speed. The paddle speed does not change in this case.

The whole AB model was implemented using the modeling and simulation software AnyLogic^®^. In the following, the simulation results are compared with experimental results.

## 4. Exemplary Application of the Agent-Based Methodology on a Rotary Press

For this case study, the effect of the change in particle size of the excipient anhydrous dicalcium phosphate in the feed is investigated. For illustration, the considerable change in particle size from one commercial quality with *x*_50_ = 167 µm to another with *x*_50_ = 64 µm and back is applied. Although changes in particle size are not expected to be as high during production as in this example, they may, however, occur due to different batches or fluctuations in the prior processes of the process chain. Due to the deformation behavior of anhydrous dicalcium phosphate, this change has a significant impact on the structural parameters as well as on the properties of the final tablet. Therefore, this case is well suited to demonstrate the capabilities of an AB simulation. In the following, the materials and the experimental setup are described.

### 4.1. Materials

**Figure 8:**
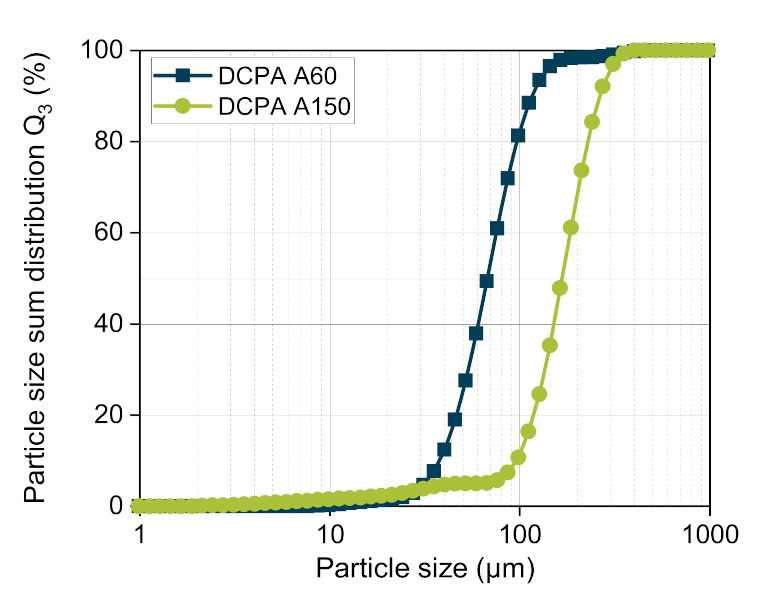
Particle size distribution of the DCPAs.

Anhydrous dicalcium phosphate (DCPA) in two grades (DI-CAFOS^®^ A60, DI-CAFOS^®^ A150, Chemische Fabrik Budenheim KG, Budenheim, Germany) were used as model materials. The characteristic particle sizes of DCPA A60 and DCPA A150, determined by laser diffraction (Mastersizer 3000, Malvern Panalytical, Kassel, Germany), differ over the whole distribution (Table 3, Figure 8). To enable their processability, DCPAs were mixed with 1 wt% magnesium stearate (MgSt, Magnesia GmbH, Lüneburg, Germany) in a cube blender (ERWEKA GmbH, Langen, Germany) for five minutes at 30 rpm. The bulk *ρ_b_* and tapped density *ρ_t_* were determined according to the Ph. Eur. 9.3 2.9.34 using a 100 mL cylinder and a volumetric analyzer (Erich Tschacher Laboratoriumsbedarf, Bielefeld, Germany). As the two grades consist of the same chemical material, the solid density *ρ_s_*, measured in triplicate with the helium pycnometer Ultrapyc 1200e (Quantachrome Instruments, Boynton Beach, FL, USA), are practically identical (Table 3).

### 4.2. Experimental Methods

The pilot scale rotary press XL 100 (Korsch AG, Berlin, Germany) was used to investigate the tablet production process. The rotary press was equipped with four flat-faced round Euro-D punches with a diameter of 11.28 mm as a mixed rotor was used. A feed frame with one rotating paddle wheel with twelve stirring blades was used. The fill cam used had a fill depth of 10 mm, while the dosing depth was set to 7 mm. The minimal punch distance for compression was set to 3.2 mm, which is subsequently an input parameter for the simulation. Since the setting on the XL 100 does not take the elastic deformation of the press itself into account, the actual punch distance during compression was determined beforehand by comparison with the results of a compaction simulator (Styl’One, Medel’Pharm, Beynost, France).

For the process experiment, the rotary press was filled to the upper end of the filling pipe with DCPA A150. A constant filling level was ensured by continuously adding new powder manually. In order to guarantee complete filling of the dies, which might be a challenge at certain process parameter settings, the paddle speed was set to 60 rpm while the turret speed was 20 rpm [47]. Tablets were taken every minute, except between minute 8–18, where tablets were taken every two minutes. After one minute, the change in particle size was induced by subsequently filling about 1 kg DCPA A60 into the filling pipe while keeping its fill level approx. constant over a period of twelve minutes. Following, DCPA A150 was filled again until the end of the experiment. The tablet weight, height and diameter were determined (*n* = 6) after 24 h. The out-die porosity as well as the tensile strength were calculated according to Fell and Netwon [48].

In order to investigate the mass fraction of the two excipients in the tablets, DCPA A60 was dyed prior to the experiments. Methylene blue was sprayed as an aqueous solution onto DCPA A60 and subsequently dried in a fluidized bed using Solidlab 2 (Syntegon Technology GmbH, Waiblingen, Germany). The tablets were dissolved in 1 M hydrochloric acid (HCl) to solve the dye, and the methylene blue content was determined by UV-Vis spectroscopy (Specord 210 Plus, Analytik Jena GmbH, Jena, Germany) at a wavelength of 669 nm. Under consideration of the dye loading of DCPA A60, the corresponding mass fraction of DCPA A60 in the tablets was calculated (*n* = 3).

### 4.3. Calibration of the Process Models

The process models presented in Section 3 were calibrated for the excipients used. Therefore, tablets (*n* = 10) were compressed at certain compression stresses (30, 50, 100, 200, 300, 400 MPa) using the compaction simulator. In order to gain the coefficients *a*, *b*, and *ρ*_0_ for the Kawakita model, the compressibility curves of the pure substances A60 and A150 were fitted in the range of 20 to 350 MPa. The starting pressure of 20 MPa was used, as the Kawakita model is generally not suitable for very low pressure ranges [49]. The coefficients can be found in Table 4.

In order to calibrate the model of Hirschberg, the densities in different states (*ρ_P,max_*, *ρ_P_*_,0_ and *ρ_out-die_*) of ten tablets of each component, DCPA A60 and A150, compressed at 30 and 400 MPa were used. The model of Ryshkewitch–Duckworth was calibrated using the tensile strengths of tablets of all six compression stresses. In order to determine the coefficients *k_b_* and *σ*_0_ and to develop a mixing rule for them, the compactability curves of the pure substances and the 50:50 blend were fitted with the model-equation (Equation (8)). The resulting progress of *σ*_0_ presents an exponential development with rising mass fraction of DCPA A150 *x_A_*_150_. Thus, for the mixtures *σ*_0_ is calculated as follows:

(9) $$\sigma_{0}=c_{1}\times e^{x_{A150}\times c_{2}}+c_{3}$$

The coefficients *c*_1_, *c*_2_, and *c*_3_ can be found in Table 5. Furthermore, *k_b_* shows a linear increase with rising mass fraction of DCPA A150 *x_A_*_150_. The value for *k_b_* can be calculated for the respective composition of the materials by Equation (10). The coefficients *m* and *n* can be found in Table 5 as well.

(10) $$k_{b}=m\times x_{A150}+n$$

### 4.4. Configuration of Simulation Setup and Error Analysis

Besides the above-mentioned agent definitions, further configurations to the AB model and model assumptions need to be made. Two events are modeled in order to change the raw material in the hopper from A150 to A60. Those time-dependent events are modeled according to the experimental set-up, i.e., the change in particle structures of the *blend in hopper* agent is scheduled after 1 min and resettled after 12 min.

The mean absolute value of the relative error *f* according to the final tablet weight between the experiment and the simulation was calculated according to Equation (11). This equation was applied for all measuring points *k* that have been made in the experiment *exp_k_* (compare Section 4.2) and those equivalent measuring points in the simulation *sim_k_*.

(11) $$f=\overset{n}{\underset{k=1}{\sum }}\frac{\left|\frac{exp_{k}-sim_{k}}{sim_{k}}\right|}{n}\forall \;k=experimental\;measuring\;points$$

## 5. Results and Discussion

### 5.1. Calibration of the Simulation Model

The sensitivity of the system towards two parameters is characterized and their calibration is necessarily conducted based on comparison with experimental data, as these parameters are not directly assessable by experiments, so far. Firstly, the effect of the theoretical diameter of the midstream, assuming a constant velocity ratio of 2:1 to the outer ring of the filling pipe, is investigated within the simulation. Secondly, the filling pattern from the feed frame fragments to the die, defining what fraction of the whole die volume is filled by successive feed frame compartments passing over the die during one filling event is also evaluated within the simulation.

**Figure 9:**
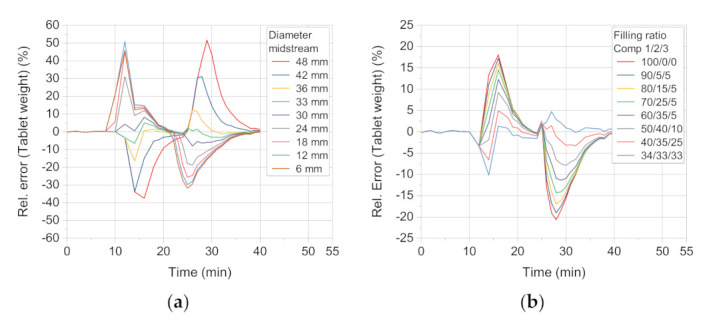
Relative error between simulation and experimental results for (**a**) different midstream diameter settings and (**b**) die filling ratio settings in relation to the tablet weight.

The sensitivity of the simulation towards deviation of the repeated midstream diameter increasing by always 6 mm shows that the relative error between the midstream diameter of 36 mm and 30 mm has the lowest relative errors (Figure 9a). Therefore, the mean of those two values (33 mm) is chosen as the midstream diameter. This proved well suited, as its *f* value of 1.32% is the lowest in this data set (see Table 6). 

The sensitivity of the distribution of filling over three compartments was studied by reducing the fraction filled by the first compartment in steps of 10%-points, distributing the rest of the filling reasonably over the second and the third compartment with the assumption that the ratio declines over the compartment number.

The lowest values are found for the ratio 40/35/25 and 34/33/33 (Table 7). This finding shows that a distribution of the die filling is better described by filling from more than one compartment of the feed frame. However, it must be born in mind that this value most likely depends on the flowability of the formulation as well as on the process parameters of turret and paddle speed. A descending pattern of 40/35/25 was used for the case study.

### 5.2. Validation of the Sub-Process Models

The behavior of the agents in the sub-processes plays an important role in the simulation, as they determine the interim results and thus the input of the subsequent sub-process models. Therefore, a careful prior validation of the underlying mathematical models is necessary and useful to investigate their accuracy and to identify possible sources of error for the simulation results. In the following, the three empirical models regarding the compressibility curve, the elastic recovery and the tensile strength are compared to experimental data examined by the compaction simulator. Subsequently, the results of the simulation are compared with the experimental data and discussed.

**Figure 10:**
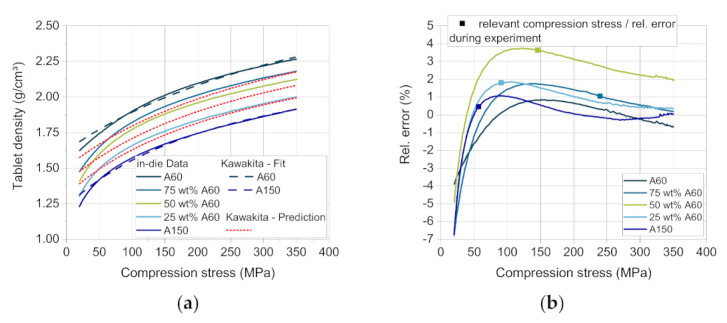
(**a**) Measured (solid lines), fitted (dashed lines), and calculated (dotted lines) compressibility curves of DI-CAFOS A60, A150, and mixtures, and (**b**) the relative error between the measured and the predicted data.

According to Busignies et al., the compressibility curves of DCPA A60 and DCPA A150 are fitted by the model of Kawakita (Figure 10a, dashed lines) to gain the material-specific coefficients *a*, *b* and *ρ*_0_. Using Equation (4) and the coefficients, the compressibility curves of the mixtures with a mass fraction of 25, 50 and 75 wt.% DCPA A60 were calculated. The comparison of the predicted and the measured curves present errors between highly negative deviation (lower values of experimental data) for all materials at a very low compression stress < 50 MPa, displaying that the lower stress range is less well described by the Kawakita model.

In the validation experiment as well as in the simulation, only specific compression stresses were reached for each blend composition, as the compression height stays approximately constant. These compression stresses and the corresponding relative errors are marked with a square (Figure 10b). The maximum positive deviation (4%) occurs for the blend with 50 wt.% DCPA A60 at about 100–150 MPa (Figure 10b), which was most likely to occur as it is the furthest from the support points of the two pure materials. The higher relative error for the blend with 50 wt.% DCPA A60 over the whole compression stress range may result from the volume-additive approach. The higher measured tablet density probably results from a volume contraction of the mixed powders in contrast to the additive volume of the single components. The small particles of DCPA A60 and their fragments may fill the pores between particles and fragments of DCPA A150, resulting in a higher tablet density than calculated. This trend can also be observed for the blends with 25 and 75 wt.% DCPA A60, although the relative error is smaller.

**Figure 11:**
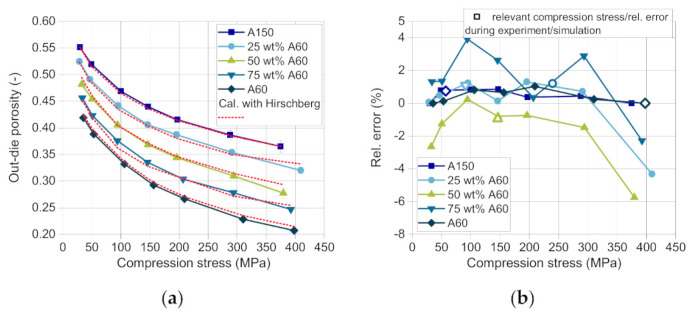
(**a**) Measured (solid lines) and calculated (dotted lines) out-die porosities of DI-CAFOS A60, A150, and mixtures, and (**b**) the relative error between the measured and the calculated data.

After the stress maximum during compaction and after ejection of the tablets, the elastic recovery takes place. As proposed by Hirschberg et al., the out-die porosity is calculated based on Equations (5)–(7) with consideration of the solid density. The relative error between the measured and the predicted out-die porosities of all substances and blends is considerably low with maximal 4% for 75 wt.% DCPA A60 and minimal −6% for 50 wt.% DCPA A60 (Figure 11b). The relevant compression stresses and the related relative errors, which occur in the validation experiment as well as in the simulation, are marked with a hollow symbol (Figure 11b).

**Figure 12:**
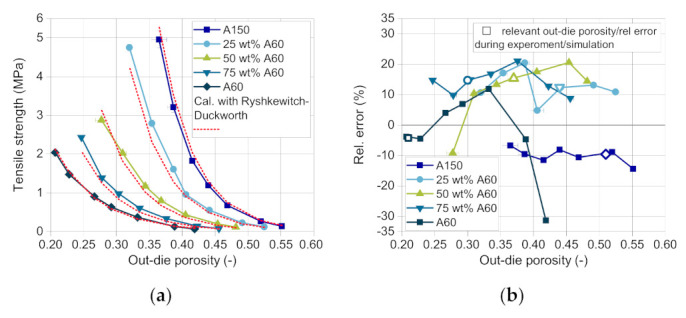
(**a**) Measured (solid lines) and calculated (dotted lines) tensile strength of DI-CAFOS A60, A150, and mixtures, and (**b**) the relative error between the measured and the calculated data.

Finally, the tablet tensile strength was estimated in dependence on the out-die porosity using the model of Ryshkewitch and Duckworth. As described in Section 4.3, a new mixing rule was developed to predict the tensile strength of tablets consisting of two different components. The coefficients of the model are calculated based on the mass fraction of DCPA A150 (Equations (9) and (10)). The compactability curves of A150 and the mixture 25 wt.% A60 (Figure 12a) show a strong increase in tensile strength with decreasing out-die porosity. With increasing mass fraction of A60, the curve flattens. The correct mathematical description of the curves is challenging due to high changes in tensile strength for low differences in the out-die porosity. This causes the high relative error between the measured and the predicted tensile strength with about −30 up to 20% (Figure 12b). Although, the calculated tensile strength curves fit the experimental values very well, as it can be seen in a first approximation in Figure 12a. Nevertheless, it has to be taken into account that the data for each composition is only relevant in a specific compression stress range and thus a porosity range, as the stress changes with the composition and so does the out-die porosity. The relevant relative errors of the tensile strength are marked for each blend composition with hollow symbols (Figure 12b). 

### 5.3. Comparison of the Simulative and Experimental Results

**Figure 13:**
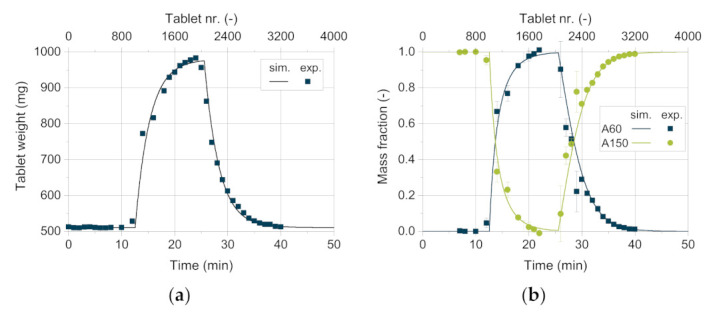
Comparison of the simulative data with experimental results for (**a**) the mass fraction of DCPA A150 and A60 and (**b**) the tablet weight.

In order to determine the quality of the AB model, the resulting CQAs obtained by the simulation are compared with the experimental results achieved from the rotary press XL 100. Figure 13 shows the tablet weight as well as the mass fraction of the respective excipient as a function of time. The tablets weigh about 500 mg at the beginning of the experiment, containing 100 wt.% DCPA A150. After about twelve minutes, the tablet weight increases significantly as the DCPA A60 content starts to rise. This can be traced back to the increasing apparent bulk density with higher DCPA A60 content as its small particles fill pores and lead to a denser arrangement of the powder bed. Therefore, the weight filled into the die increases, resulting in a higher tablet weight. With increasing mass content of DCPA A60, the change in mass fraction as well as tablet weight flattens. Due to the residence time distribution of DCPA A150, it takes a certain time to fill remaining DCPA A150 particles into the dies, leading to change in content inside the feed frame.

The good agreement between the simulative data and the measured values for the tablet weight and mass fraction (Figure 13) are particularly worth to mention. Only for high DCPA A60 mass fractions, simulative data exhibit slightly lower tablet weights compared to the measured values. This underestimation might be linked to die filling, where apparent densities above the bulk density are possible due to particle rearrangements during forced feeding and especially during dosing [47]. To meet the tablet weight, obtained by the experiment, an apparent consolidated bulk density after filling of 0.73 g/cm^3^ had to be used, which is considerably higher than the bulk density of 0.68 g/cm^3^. As described in the literature, good flowing powder as dicalcium phosphate consolidates at high paddle speeds and low turret speeds inside the die. The process parameters selected here as well as the material properties support this hypothesis. The same observations were made for DCPA A60, so the apparent consolidated bulk density after filling was taken to be 1.41 g/cm^3^ compared to the bulk density of 1.33 g/cm^3^.

Although no specific mixing model is introduced in the AB simulation, mixing and the distribution of the newly entering DCPA A60 can be represented by the set-up of the simulation itself. As described in Section 3, the midstream with a diameter of 33 mm simulates a flow profile inside the filling pipe according to its geometry, by having double the velocity as the surrounding powder flow (compare Section 3). Thus, a powder flow similar to the actually expected is aspired. As soon as the powder, represented by the *blend in filling pipe*, enters the feed frame, the mass fraction of the respective compartment is recalculated. Therefore, the twelve compartments work as independent continuous stirred tank reactors (CSTRs), as the mixing ratio is constant within the entire compartment in each time step. Comparing this setting to the visual observations on the tablet press using a transparent feed frame, a completely homogenous concentration of the excipients is not realistic, especially not directly after new powder with a different composition entered the feed frame. Mixing in the feed frame over time can be observed, while the powder is not only mixed inside an interspace but is also able to change the compartment if the particles are close to the bottom or flow over the paddles in real experiments. Due to the high paddle speed of 60 rpm and the simultaneously low turret speed of 20 rpm, leading to a high residence time, the powder can mix quickly inside the feed frame. Puckhaber et al. investigated the residence time distribution on the XL 100 for a pure dicalcium phosphate with similar powder properties as DCPA A150, showing strong intermixing for the process parameters used in this study [50]. This improves the model quality in so far that the simulated mass fraction, resulting from the compartments, modeled as CSTRs, is in good agreement with the experimental data. In future work different combinations of paddle and turret speed shall be looked at to investigate whether the concept of the CSTRs is still valid or must be refined for combinations of free flowing with poorly flowing materials, for example.

For the experimental data of the mass fraction, a very good correlation between experimental and simulative values was found (Figure 13b). However, higher standard deviations are observed for the experimentally determined mass fraction than for the tablet weight, which shows standard deviations below 1.5%. The mass fraction is determined by using dyed DCPA A60, which intrinsically shows higher standard deviations of the loading as ten samples à 100 mg presented a relative standard deviation of 11.7%. Therefore, the tablet weight (*n* = 6) is a more reliable and more facile to determine parameter to compare the simulative and experimental data.

**Figure 14:**
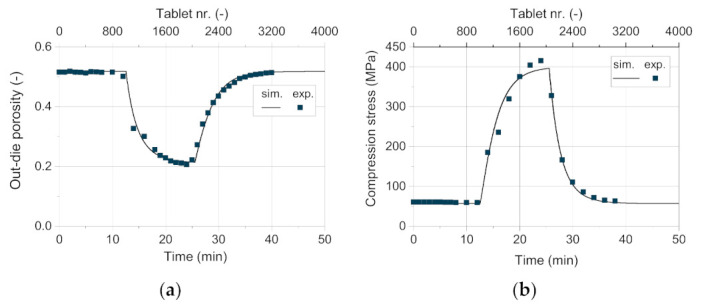
Comparison of the simulative data with experimental results for (**a**) the out-die porosity and (**b**) the compression stress.

Besides the tablet weight, the out-die porosity and the tensile strength are of particular interest regarding their possible correlation to disintegration time and the handling stability of the tablets, respectively. The out-die porosity exhibited by the simulation fits very well with the experimental data (Figure 14a). To achieve this degree of convergence, the models used to describe the maximum compression stress and the total elastic recovery as function of filling weight and blend density do present a very good applicability. 

**Figure 15:**
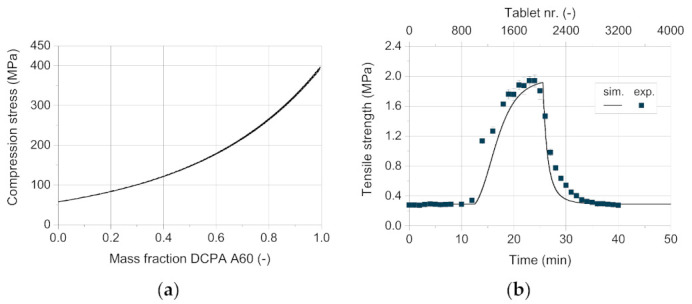
(**a**) Calculated compression stress over the mass fraction of DCPA A60. (**b**) Comparison of the simulative data with experimental results for the tensile strength.

As shown for the validation of the process models, the in-die tablet density and thus the in-die tablet porosity have a deviation between −2 and 4% above 50 MPa (Figure 10b). As the tablets contain only DCPA A150 at a compression stress of about 60 Mpa in this case, the apparent error due to the Kawakita model is close to 0%. As Figure 15a shows, the resulting simulated compression stress in this study for 75 wt.% DCPA A60 is around 200 Mpa. In this stress range, the deviation for the respective content is small (Figure 11b, hollow symbol). Although tablets with 50 wt.% and 25 wt.% DCPA A60 present high relative errors around 400 Mpa (Figure 11b), they do not affect the simulation results as the apparent compression stress is lower than 150 Mpa for these mass fractions. Therefore, the total mean error for the out-die porosity is low with 2.13% as listed in Table 8. 

For the comparison of the experimentally recorded compression stress on the rotary press and the simulated values, a very good match can be observed (Figure 14b). In the high stress range, the simulative data underestimates the experimental values. This might be due to the slightly lower simulated tablet weight at high DCPA A60 content. At such a high compression state, a low change in weight has a relatively strong effect on the corresponding compression stress (compare Figure 10a). For further research, the consolidation of the powder during and after filling has to be addressed in more detail to better calculate the tablet weight and thus predict the compression stress even more accurately.

**Figure 16:**
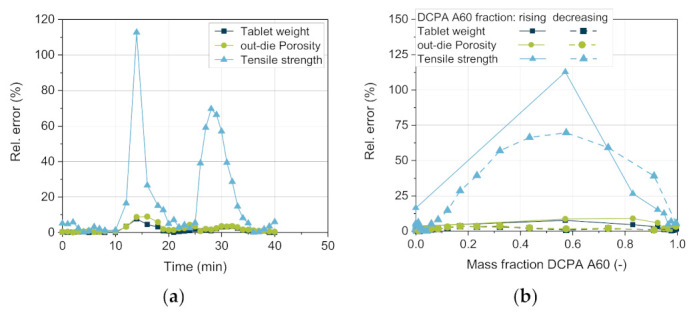
Relative error of the simulative data compared to experimentally determined values for the (**a**) tablet weight, out-die porosity, and tensile strength over time and (**b**) the mass fraction of DCPA A60.

Regarding the tensile strength, considerably higher deviations between the simulative and the experimental data are observed (Figure 15b). While the measured values match well for pure DCPA A150 and pure A60, the correct prediction of the tensile strength during the change in content is challenging (Figure 16). The tensile strength obtains the highest relative errors for the simulative data with over 100% with rising mass fraction of DCPA A60 and about 70% with decreasing content (Figure 16a). The lower quality of the predicted data of the tensile strength can be traced back to the model and especially the mixing rule used. For the model validation, already relative errors of -35 up to 20% are obtained, while no specific trend for the mass fraction is observable (Figure 12b). Nevertheless, the model of Ryshkewitch uses the out-die porosity as input parameter to calculate the corresponding tensile strength, which includes previous errors. Due to the strong increase in strength for low changes in porosity, small differences in the porosity have a high impact. A parabolic shape of the relative error over the mass content can be observed, which indicates a systematic error for the determination of the tensile strength (Figure 16b).

Interestingly, comparing the increasing and decreasing DCPA A60 fractions, the relative errors of all three parameters present higher values with increasing DCPA A60 fraction than vice versa (Figure 16b). This might be due to the change in the apparent bulk density as the actual volume of the powder is lower than the calculated one by adding the respective volumes of each powder. Additionally, the higher deviation with rising DCPA A60 content in comparison to the decreasing content can indicate different residence time distributions and thus, different distribution profiles of the two excipients. Both will be investigated in future research.

Considering the overall accuracy of the simulation results, the mean absolute relative error *f* (compare Equation (11)) of the CQAs can be seen in Table 8.

Besides the tensile strength of the tablets, all other CQAs have a mean relative error of less than or equal to 6.99%. However, the quality of the AB model can only be as good as the quality of the physical models used to calculate product properties. For the calculation of the tensile strength a better model and, moreover, a better bulk density determination of the component mixture is necessary.

From a simulation point of view, the AB modeling approach allows the observation of each agent over the complete simulation time. Furthermore, the input materials have been digitally marked and allow a tracking and tracing over the whole process. This fact allows to investigate the output in relation to the input on a specific matter. With this, several processing questions can be answered, e.g., the characterization of tablets that have fractions of a specific input material. This shows the potential of AB models to be capable of model particle-based processes chains at low computational cost, prospectively making their application in real time control of pharmaceutical production processes feasible.

## 6. Conclusions and Outlook

This study presents the AB simulation approach, which is, to the best knowledge of the authors, for the first time applied for the simulation of a pharmaceutical, particle-based process chain. The implementation of such an AB simulation framework necessitates a careful analysis of the individual process steps to derive simple but effective sub-models. Mindful assumptions are necessarily defined and calibrations of the systems must be performed to implement relations that are not directly assessable by experimental means. The results of the simulation compared with experimental data regarding several CQAs were predicted predominantly with only minor errors. It is shown that an AB modeling approach is an alternative to the classical simulation paradigm for solids simulation, combining the positive characteristics of the FSS and the DEMs, i.e. the ability to model process chains or sub-process chains and the representation of material-process interactions, respectively. As is known regarding FSSs, physical sub-models of high quality are required to describe the resulting product properties as a function of process time exactly.

Further research in this field should consider the effect of additional materials with different properties as well as predecessor and successor steps, e.g., blending, granulation and coating. The main research questions consider the interconnection between these models and the transfer of deep process knowledge into an AB simulation approach. This model development is by now rather complex and further modeling strategies should be explored. Moreover, the agent’s behavior should be enhanced to foster better and more accurate simulation results. With this, several effects, as for example the segregation of components, can be modeled within an AB model. Furthermore, environmental influences should be integrated in AB models, e.g., the environmental relative humidity or temperature.

In order to contribute to the digitalization of manufacturing systems, the resulting AB process chain models need to be empowered for process control, e.g., within cyber-physical production systems. To this end, the development of an AB model can be based on or assisted by QbD approaches and use inline data by coupling PAT with the AB model to allow the forecast of product quality over complex process chains close to real time. Additionally, the design space for new products might be more easily defined. New formulations can already be evaluated in advance using the simulation before further experiments are conducted, which saves time and costs. By now, the simulation approach is able to perform descriptive as well as diagnostic analytics and in addition is much faster than the experimental process. In future research, this model needs to enable predictive or even prescriptive analytics. This will allow future manufacturing systems not to be mandatorily centralized and bound to the necessary process knowledge but will be able to work in decentralized locations with simulation models controlling the processes.

## Figures and Tables

**Table 1:** Dynamic FSS modeling and simulation approaches to pharmaceutical tableting.

Authors	Considered Process Scope			Considered CQA for Tableting Process
	Blending	Granulation	Tableting	
Boukouvala et al., 2012	X	X	X	▪ Tablet porosity ▪ Composition of material ▪ Relative standard deviations of input materials
Boukouvala et al., 2013	X	X	X	▪ Tablet dissolution ▪ Particle size distribution
Rogers et al., 2013	X		X	▪ Tablet hardness ▪ Friability ▪ Composition of material ▪ Dissolution performance

**Table 2:** Overview of the states of rotary press process agents, their behaviors, and their interactions.

Agent	State	Behavior	Interactions	Calculations
Hopper	Fill filling pipe	Determine/specify materials for the transmission in filling pipe	Filling the filling pipe with blend from hopper	
Filling pipe	Movement in filling pipe	Blend in pipe is transported towards feed frame, realize flow profile inside the pipe	Triggering hopper to fill voids	Material fractions
	Fill feed frame		Filling the feed frame compartments with blend in filling pipe	Material fractions
Feed frame	Turning	Turning the compartments according to paddle speed	Feed die with blend and triggering filling pipe	
	Die filling		Filling of die out of different feed frame compartments	Material fractions
				Mixed density (Equation (1))
				Tablet weight (Equation (2))
Die (and punches)	Compaction	Applying the compression stress on the powder blend according to minimal in-die height *h_min_* under consideration of the elastic deformation of the tablet press	Creating a stress based on product agent properties, creating tablet, triggering feed frame	Compression stress (Equations (3) and (4))
				Tablet in-die density (Equation (3))
	Ejection	Waiting according to turret speed		Tablet out-die porosity (Equations (5)–(7))
				Tablet tensile strength (Equation (8))

**Table 3:** Characteristic particle sizes, solid, bulk, and tapped density for DCPA A60 and A150.

Material	*x*_10_ (µm)	*x*_50_ (µm)	*x*_90_ (µm)	*ρ_s_* (g/cm^3^)	*ρ_b_* (g/cm^3^)	*ρ_t_* (g/cm^3^)
DCPA A60	34	64	116	2.849	1.33	1.51
DCPA A150	96	167	263	2.842	0.68	0.75

**Table 4:** Coefficients for the Kawakita model.

Excipient	*ρ*_0_	*a*	*b*	*R*²
DCPA A150	1.22954	0.4981	0.0072	0.9962
DCPA A60	1.62302	0.4891	0.0041	0.9959

**Table 5:** Coefficients to calculate *σ*_0_ and *k_b_*.

Coefficients to Determine *σ*_0_		Coefficients to Determine *k_b_*	
*c*_1_	19.99	*m*	3.80
*c*_2_	5.56	*n*	15.09
*c*_3_	28.32		

**Table 6:** Relative error of midstream model configuration regarding the tablet weight.

Midstream Diameter (mm)	42	36	33	30	24	18	12	6
Mean Absolute Value of the Relative Error (*f*) (%)	6.02	1.86	1.32	2.23	5.19	7.27	8.82	9.05

**Table 7:** Relative error of die filling ratio configuration regarding the tablet weight.

Filling ratio (%)	1. Compartment	100	90	80	70	60	50	40	34
	2. Compartment	0	5	15	25	35	40	35	33
	3. Compartment	0	5	5	5	5	10	25	33
Mean Absolute Value of the Relative Error (%)		5.35	5.04	4.64	4.11	3.38	2.46	1.32	1.24

**Table 8:** Final agent-based simulation result deviations of critical quality attributes.

Critical Quality Attributes		Tablet Weight	Mass Fraction	Out-Die Porosity	Tensile Strength
Mean absolute relative error (*f*) (%)		1.34	6.99	2.13	17.69
Deviation (%)	Max	7.60	43.82	8.93	112.74
	Upper quartile	2.15	9.34	3.25	27.06
	Median	0.83	2.41	1.55	5.21
	Lower quartile	0.20	0.00	0.63	2.38
	Min	0.00	0.00	0.02	0.00

## Data Availability

Not applicable.
